# Comparing Postpartum Estimated and Quantified Blood Loss Among Racial Groups: An Observational Study

**DOI:** 10.7759/cureus.25299

**Published:** 2022-05-24

**Authors:** Daniel Katz, Shradha Khadge, Brendan Carvalho

**Affiliations:** 1 Anesthesiology, Perioperative and Pain Medicine, Icahn School of Medicine at Mount Sinai, New York, USA; 2 Anesthesiology, Perioperative and Pain Medicine, Stanford University Medical Center, Stanford, USA

**Keywords:** ethnic disparities, maternal morbidity, postpartum hemorrhage, racial disparities, quantified blood loss

## Abstract

Objective: Racial and ethnic disparities in peripartum blood loss and postpartum hemorrhage (PPH) have not been adequately evaluated. We sought to compare postpartum blood loss and PPH in African American and Hispanic parturients compared to other groups.

Methods: This was a secondary analysis of an observational study at a tertiary academic center of a historical (August 2016 to January 2017) and interventional (August 2017 to January 2018) cohort of 7618 deliveries. Visual estimation of blood loss (EBL) was used in the historical group and quantitative blood loss (QBL) was implemented in the intervention group. Our primary endpoint was median blood loss in African Americans versus other racial groups between cohorts.

Results: A total of 7618 deliveries were evaluated; 755 (9.9%) were identified as African American with 1035 (13.6%) identifying as Hispanic. Blood loss was similar in racial groups using EBL (p=0.131), but not QBL that was 430 (227-771) in African Americans and 348 (200-612) in non-African Americans (p<0.001). PPH was greater among African Americans in both groups (10.3% vs. 6.9% in EBL cohort, p=0.023, and 16.9% vs. 11.6% in QBL cohort, p<0.001).

Conclusion: Racial and ethnic differences in peripartum blood loss were more apparent with QBL than EBL. It is unknown if these differences are caused by provider cognitive bias, socioeconomic differences, language barriers and/or other factors.

## Introduction

Racial and ethnic disparities in maternal outcomes are well described. Pregnancy-related mortality and morbidity for African Americans is several fold higher compared to Caucasian women [1-6]. Strikingly, pregnancy-related mortality and morbidity remains significantly elevated even after adjusting for patient characteristics and comorbidities [1]. These disparities and increased risk are also described for Hispanic parturients, and persist even after controlling for patient characteristics and socioeconomic and educational differences [6-9].

Postpartum hemorrhage (PPH) is one of the most common causes of preventable maternal morbidity and mortality [10-14]. Black and Hispanic women have an increased risk of PPH and are at a higher risk of morbidity and mortality following hemorrhage compared to Caucasians [4,5,9,15-20]. This racial disparity remains even after adjusting for patient characteristics, comorbidities, socioeconomic status, and delivery hospital [15]. Differences in PPH and blood loss have traditionally been evaluated using visual estimation of blood loss (EBL), which have been found to be inaccurate [21-23]. The amount and distributions of blood loss are different when blood loss is quantified (quantitative blood loss or QBL) and not visually estimated. In several previous studies, quantifying blood loss using the Triton QBL system (Gauss Surgical, Inc. Menlo Park, CA) was associated with a higher and more realistic wider range of blood loss compared to a narrow range in the subjective visual estimate [24-26]. Using QBL also improved early recognition and treatment of peripartum hemorrhage, and many institutions are transitioning to using a QBL system as maternal obstetric safety organizations have suggested in their PPH bundles [14,24,27]. Most studies of racial and ethnic differences in peripartum blood loss and incidence of PPH use an International Statistical Classification of Diseases and Related Health Problems (ICD) code for hemorrhage or blood transfusions or documented EBL, but to our knowledge no studies have looked at racial and ethnic disparities with QBL [15,17,19,20].

In this study, we aimed to evaluate racial disparities surrounding postpartum blood loss and PPH, especially, when blood loss is quantified compared to when it is visually estimated. Using a standardized metric for quantification of blood loss (QBL) as opposed to visual EBL may provide a more accurate look at differences in postpartum blood loss between groups. The primary outcome was the difference in the median QBL blood volume loss in African Americans compared to other racial groups. As QBL is less subjective, we hypothesize that postpartum blood loss and PPH rates will differ along racial and ethnic divides depending on whether blood loss was measured by visual EBL versus measured QBL. This would likely be due to implicit bias present in visual measurements as well as cognitive bias of observers.

## Materials and methods

This study is a secondary analysis of a large observational cohort study conducted at a single academic medical center (Icahn School of Medicine at Mount Sinai, New York) and was approved by the Mount Sinai Health System Institutional Review Board (protocol #16-00976) [24]. This manuscript adheres to the appropriate Strengthening the Reporting of Observational studies in Epidemiology (STROBE) guidelines. Informed consent was waived for this system-wide clinical care initiative. On April 2 2017, the Mount Sinai Hospital System began implementing a protocol using the Triton system to quantify blood loss for all parturients at delivery, with continuation in the postpartum wards.

In cesarean deliveries (CDs), the Triton colorimetric system utilizes a camera to analyze surgical sponges and canisters in the operating room and applies an algorithm to estimate the amount of hemoglobin in the sponge and canisters. This system has been extensively tested and has a high degree of correlation with hemoglobin extraction methods used in a laboratory. The patient’s hemoglobin concentration is entered into the system and a volume of blood is calculated. This value is obtained prior to entry into the operating room from prior values. In emergency cases, a default value can be entered and then modified once a value is obtained. Since the system will measure hemoglobin in total, it is resistant to hemodilution. However, during a hemorrhage it is considered best practice to update the hemoglobin value of the patient such that the volume of blood loss remains accurate. Gravimetric analysis is used to quantify blood on items that cannot be scanned using the colorimetric system (e.g. sheets, drapes). All objects that are visibly soiled with blood are placed onto the Triton system’s smart scale. The user then enters the items that are on the scale and the system subtracts the known dry weights of those items into an algorithm that quantifies blood loss. These two systems are used in conjunction to produce a QBL during a CD. The QBL monitoring continues in the recovery room and in the postpartum wards using only the gravimetric QBL analysis.

In vaginal deliveries (VDs), a calibrated v-drape is used for volumetric analysis. Immediately following amniotomy, the amniotic fluid volume is noted in the v-drape and entered into the system. After a VD, the initial amniotic fluid volume is subtracted from the total fluid volume in the v-drape and used to determine blood loss. In addition, gravimetric analysis was used on all objects visibly soiled with blood as described above. The QBL monitoring continued after delivery in the labor room and in the postpartum wards using only the gravimetric analysis. The blood loss during the intra- and postpartum periods were combined to obtain the total QBL. For further details on the methodologies for quantifying blood loss, please see the original study [24].

**Figure 1:**
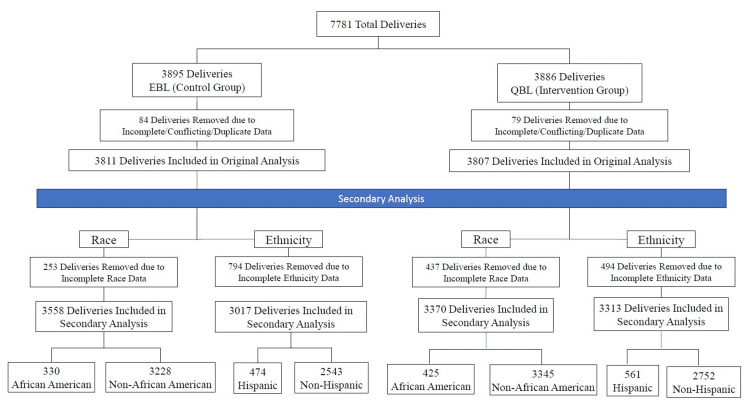
Patient flow diagram EBL, estimation of blood loss; QBL, quantitative blood loss

The intervention cohort data were collected during a six-month period between August 1, 2017, and January 31, 2018. To account for seasonal differences, a control cohort was selected from delivery data in the same period of time in the year prior, i.e., August 1, 2016 to January 31, 2016. Unique visit numbers were used to eliminate deliveries of multiples and duplicate medical record entries. In the original study, out of 7781 charts that were queried, 163 charts were excluded for missing or duplicate data [24]. In this study, we looked at the same 7618 charts and then excluded those patients with missing racial or ethnic data for the final analysis (Figure 1).

Patient demographics including age, race, ethnicity, body mass index, gravidity, parity, gestational age, and mode of delivery were extracted from the electronic medical records. Race and ethnicity at our center is self-reported. A hemorrhage risk score was calculated using our institutional scoring system (Table 1). Low-risk patients had no known risk factors. Medium-risk patients had one of the following risk factors: prior cesarean delivery, prior uterine surgery, prior laparotomies, multiple gestations, multiparity, prior postpartum hemorrhage, large myomas, morbid, obesity or estimated fetal weight >4000 g. Patients were considered high risk if they had placenta previa, suspected placenta accreta, platelet count <70,000, active bleeding, coagulopathy or if two or more medium hemorrhage risk factors were present.

**Table 1:** Hemorrhage risk factor assessment

Risk level	Risk factor
High-risk factors	Placenta previa/low-lying placenta, suspected accreta/percreta, platelet count <70,000, active bleeding, coagulopathy
Medium-risk factors	Prior cesarean delivery, prior uterine surgery, prior laparotomies, multiple gestation, >4 prior births, prior postpartum hemorrhage, large myomas, estimated fetal weight >4000 g, morbid obesity
Low-risk factors	Hematocrit <30%, none of the above factors
Level determination	High risk (3): any high-risk criteria, or two or more medium-risk criteria; medium risk (2): one medium-risk criteria; low-risk (1): no medium- or high-risk criteria

In the control cohort, visual EBL was agreed upon by the obstetrician, nursing staff and anesthesiologist (when present). These data were compared to the intervention cohort where the QBL was obtained from the Triton system as described above. Both cohort data were obtained retrospectively. During the study time periods, there were no major changes to caseload or staffing. Practitioners continued all routine care and procedures during the study period other than the implementation of the Triton QBL system and associated practice changes. These practice changes included the following: (1) postpartum ordering of blood tests was only required if the patient had greater than normal blood loss during delivery, i.e., >600 mL for VD and >1000 mL for CD and (2) the clinical care team on the postpartum ward triaged which patients were escorted to the bathroom based on a measured QBL of >600 mL for VD and >1000 mL for CD. There was no defined protocol for blood administration. For the purposes of data analysis, PPH was defined as blood loss greater than 1 L.

Main outcome measures

The aim of this secondary analysis was to investigate the median blood loss volumes in racial and ethnic groups in each cohort. Specifically, our primary outcome was to compare QBL blood loss values in African American and African parturients as compared to non-African American and non-African parturients between the EBL and QBL cohorts. Exploratory and secondary analyses included incidence of PPH (defined as blood loss >1 L), rate of CD, high hemorrhage risk score differences between groups as well as an investigation into differences in ethnicities defined as Hispanics versus non-Hispanic. At Mount Sinai, racial and ethnic designations are self-reported and patients have the option to decline to answer these questions. Patients who declined to answer were excluded from the analysis. For the purposes of the study, race and ethnicity were considered to be binary by reporting. The two groups for race were African American and African parturients compared to non-African American and non-African parturients. For ethnicity, it was Hispanic parturients compared to non-Hispanic parturients.

Statistical analysis

All variables were tested for normality using the Shapiro-Wilk test. Normally distributed variables are reported as mean (standard deviations [SDs]) with non-normally distributed variables as median (interquartile range [IQR]). Two-tailed t-tests were used to compare normally distributed continuous variables, while non-parametric tests such as the Mann-Whitney U test were used for non-normal variables. Categorical variables were tested using the chi-square method. For our primary outcome, the median blood loss between racial groups, a difference was considered significant if the p-values were less than 0.05. The same threshold was used for secondary endpoints, but it should be noted that these are only exploratory in nature. To examine the association between variables and blood loss between groups, multivariable linear regression was utilized. Statistics were performed in IBM SPSS Statistics, version 24 (IBM Corp., Armonk, NY).

## Results

Of the 7618 deliveries originally evaluated, 755 (9.9%) of parturients were African/African American with 1035 (13.6%) of patients identifying as Hispanic. There were 290 patients missing race and 1288 patients missing ethnicity. These patients were excluded from this analysis (Figure 1).

For all deliveries, the distribution of blood loss was similar between African American/African and non-African American/African groups using visual EBL (p=0.131), but African/African Americans had significantly higher blood loss when using QBL (p<0.001). The percentage of patients with PPH was greater among African/African Americans in both groups (EBL, p=0.023; QBL, p<0.001). African/African Americans had significantly higher rate of CD compared to non-African/African Americans in both cohorts (37.9% vs. 31.6% in the EBL cohort, p=0.019, and 44.5% and 31.7% in the QBL cohort, p<0.001). There was no difference in our institutional high hemorrhage risk score between racial groups in either cohort (Table 2).

**Table 2:** Blood loss information by race (African American/African vs. non-African American/African) in the EBL and QBL study cohorts EBL, estimated blood loss; QBL, quantitative blood loss; IQR: interquartile range; PPH, postpartum hemorrhage; AI, artificial intelligence; L&D, labor and delivery QBL assessment was done using Triton AI and L&D. *Placenta previa, suspected accreta, platelet count <70,000, active bleeding, coagulopathy or two or more medium-risk criteria including prior cesarean delivery, prior uterine surgery, prior laparotomies, multiple gestations, multiparity, prior postpartum hemorrhage, large myomas, morbid obesity, estimated fetal weight >4000 g

Variables	African American/African EBL cohort, n=330	Non-African American/African EBL cohort, n=3228	p-value		African American/African QBL cohort, n=425	Non-African American/African QBL cohort, n=3345		p-value
Median blood loss (mL), median (IQR)	350 (300-800)	350 (300-800)		0.131	430 (227-771)	348 (200-612)	<0.001	
Patients with PPH, n (%)	34 (10.3)	223 (6.9)		0.023	72 (16.9)	389 (11.6)	0.002	
High hemorrhage risk score*, n (%)	16 (4.8)	150 (4.6)		0.734	14 (3.3)	159 (4.8)	0.247	

Similar to racial divides, when looking at the data based on ethnicity, blood loss distributions were not different in the visual EBL cohort (p=0.951), whereas Hispanic compared to non-Hispanic women had significantly higher blood loss in the QBL cohort (p=0.049). The diagnosis of PPH in the EBL cohort was similar between Hispanic and non-Hispanic women (p=0.471), but Hispanic women had significantly higher rates of diagnosed PPH in the QBL cohort (p=0.018). Hispanics had a significantly higher rate of CDs compared to non-Hispanics in the EBL cohort (36.5% vs. 31%; p=0.019), but not in the QBL cohort (34.6% vs. 33.2%; p=0.531). There was no difference in our institutional high hemorrhage risk score between ethnic groups in the EBL cohort (p=0.124), but it did differ in the QBL cohort (p=0.001; Table 3).

**Table 3:** Blood loss information by ethnicity (Hispanic vs. non-Hispanic) in the EBL and QBL study cohorts EBL, estimated blood loss; QBL, quantitative blood loss; IQR: interquartile range; PPH, postpartum hemorrhage; AI, artificial intelligence; L&D, labor and delivery QBL assessment was done using Triton AI and L&D. *Placenta previa, suspected accreta, platelet count <70,000, active bleeding, coagulopathy or two or more medium-risk criteria including prior cesarean delivery, prior uterine surgery, prior laparotomies, multiple gestations, multiparity, prior postpartum hemorrhage, large myomas, morbid obesity, estimated fetal weight >4000 g

Variables	Hispanic EBL cohort, n=474	Non-Hispanic EBL cohort, n=2543	p-value	Hispanic QBL cohort, n=561	Non-Hispanic QBL cohort, n=2752	p-value
Median blood loss (mL), median (IQR)	350 (300-800)	350 (300-800)	0.951	379 (210-705)	350 (200-624)	0.049
Patients with PPH, n (%)	36 (7.6)	170 (6.7)	0.471	83 (14.8)	310 (11.3)	0.018
High hemorrhage risk score*, n (%)	16 (3.4)	131 (5.2)	0.124	10 (1.8)	143 (5.2)	0.001

To further delineate the impact of race on blood loss at delivery, a multivariable regression model was created for each group with the following inputs as confounders: gravidity, parity, maternal age, maternal BMI, fetal weight, admission hemorrhage risk score, multiple gestations, delivery type (VD vs. CD) and African American race. The complete models are seen in Table 4. Of significance, in the EBL cohort, the African American race was not predictive of blood loss with a beta coefficient of 4.3, significance of 0.705 with a 95% CI of -17.9 to 26.5. In the QBL cohort, the African American race was associated with higher blood loss with a beta coefficient of 35.9, significance of 0.023 and a 95% CI of 4.8 to 67.0.

**Table 4:** Multivariable regression model for delivery blood loss stratified by EBL and QBL and African American race EBL, estimated blood loss; QBL, quantitative blood loss; AI, artificial intelligence; L&D, labor and delivery; BMI, body mass index QBL assessment was done using Triton AI and L&D. *Placenta previa, suspected accreta, platelet count <70,000, active bleeding, coagulopathy or two or more medium-risk criteria including prior cesarean delivery, prior uterine surgery, prior laparotomies, multiple gestations, multiparity, prior postpartum hemorrhage, large myomas, morbid obesity, estimated fetal weight >4000 g

Cohort	Variable	Beta coefficient	p-value	95% CI
EBL	Maternal age	1.21	0.035	0.08 – 2.35
	Maternal BMI	0.845	0.189	-0.41 – 2.35
	Gravidity	-4.52	0.025	-8.46 – -0.58
	Parity	-5.69	0.061	-11.65 – 0.26
	Gestational age	0.382	0.867	-4.09 – 4.85
	Hemorrhage risk score*	25.88	0.000	12.02 - 39.74
	Fetal weight	0.02	0.016	0.00 – 0.03
	Multiple gestation	156.47	0.000	116.27 – 196.68
	African American race	6.35	0.577	-15.95 – 28.65
QBL	Maternal age	0.22	0.802	-1.52 – 1.96
	Maternal BMI	1.35	0.161	-0.54 – 3.25
	Gravidity	-1.43	0.664	-7.8 – 5.03
	Parity	-34.90	0.001	-44.41 – -25.39
	Gestational age	-7.27	0.053	-14.65 – 0.102
	Hemorrhage risk score*	38.93	0.000	17.41 – 60.45
	Fetal weight	0.09	0.000	0.06 – 0.11
	Multiple gestation	270.03	0.000	205.07 – 334.99
	African American race	36.64	0.021	5.50 – 67.78

When race was substituted for ethnicity, the model changed. In the EBL cohort, Hispanic ethnicity was associated with blood loss with a beta coefficient of 0.170, a significance of 0.026 and a 95% CI of 0.02 to 0.31. In the QBL cohort, Hispanic ethnicity was not associated with blood loss with a beta coefficient of 0.081, a significance of 0.582 and a 95% CI of -0.206 to -0.368 (Table 5).

**Table 5:** Multivariable regression model for delivery blood loss stratified by EBL and QBL and Hispanic ethnicity EBL, estimated blood loss; QBL, quantitative blood loss; BMI, body mass index; AI, artificial intelligence; L&D, labor and delivery QBL assessment was done using Triton AI and L&D. *Placenta previa, suspected accreta, platelet count <70,000, active bleeding, coagulopathy or two or more medium-risk criteria including prior cesarean delivery, prior uterine surgery, prior laparotomies, multiple gestations, multiparity, prior postpartum hemorrhage, large myomas, morbid obesity, estimated fetal weight >4000 g

Cohort	Variable	Beta coefficient	p-value	95% CI
EBL	Maternal age	1.17	0.034	0.08 – 2.26
	Maternal BMI	0.90	0.136	-0.28 – 2.09
	Gravidity	-3.91	0.043	-7.77 – -0.12
	Parity	-6.18	0.036	-11.96 – -0.415
	Gestational age	1.15	0.602	-3.17 – 5.47
	Hemorrhage risk score*	26.48	0.000	13.19 – 39.76
	Fetal weight	0.01	0.044	0.00 – 0.031
	Multiple gestation	143.64	0.000	105.99 – 181.30
	Hispanic ethnicity	0.17	0.026	0.02 – 0.319
QBL	Maternal age	0.02	0.982	-1.70 – 1.74
	Maternal BMI	1.82	0.053	-0.24 – 3.67
	Gravidity	-0.89	0.785	-7.31 – 5.53
	Parity	-35.45	0.000	-44.87 – -26.02
	Gestational age	-6.2	0.094	-13.57 – 1.06
	Hemorrhage risk score*	38.65	0.000	17.33 – 59.97
	Fetal weight	0.087	0.000	0.06 – 0.11
	Multiple gestation	268.82	0.000	204.29 – 333.36
	Hispanic ethnicity	0.081	0.582	-0.20 – 0.36

## Discussion

In this secondary analysis of a large observational study, we found that both racial and ethnic differences in blood loss distributions and incidences of PPH depended on whether blood was assessed subjectively using visual EBL or objectively using QBL. When using a standardized QBL measurement, African Americans/Africans had significantly more peripartum blood loss than non-African Americans/Africans. The data however show less differences along racial and ethnic divides when using visual EBL. To our knowledge, this observation has not been shown before.

The previous literature has shown an increase in peripartum blood loss, and higher risk of morbidity and mortality following hemorrhage in Black and Hispanic parturients [4,5,9,15-20]. However, these studies often defined hemorrhage using a medical diagnosis code for hemorrhage or documented EBL [15]. Our study confirmed these racial and ethnic differences with QBL measurements confirming these previously described disparities. Interestingly, we did not show significant differences between racial or ethnic groups in the EBL cohort. Studies have shown that EBL is subject to inaccuracies and many potential biases [21-23]. Subjective visual EBL is more prone to human error and cognitive biases that may have been masking the underlying disparities that were demonstrated in the QBL cohort. The objective nature of the QBL measurements would eliminate many potential biases.

Maternal safety organizations have suggested using QBL as part of their PPH bundles and optimal stage-based obstetric hemorrhage management [14,27]. Using an objective QBL system should help recognize and treat hemorrhage earlier [24]. This may lessen the burden of morbidity and mortality following peripartum hemorrhage to which racial and ethnic minorities are more susceptible [4,9,17-20]. Surprisingly, the institutional hemorrhage risk scores in African American/African and Hispanic parturients were not different suggesting that differences in observed blood loss and PPH between groups were not due to more conditions associated with obstetric hemorrhage, but rather differences in peripartum events in these racial and ethnic groups. One maternal safety consensus bundle provided a framework to reduce peripartum racial and ethnic disparities by first improving measurement of disparities, recognizing and educating providers on implicit and institutional bias and providing interpreters for the patients’ preferred language [28].

The rate of CDs was higher in African Americans in both EBL and QBL cohorts in this study, similar to other studies [29,30]. A prior study of a large-scale quality improvement hemorrhage bundle has suggested that decreasing the rate of CDs in this group may decrease hemorrhage [28]. The higher CD rate may have contributed to the observed racial disparities seen in blood loss and PPH in both the QBL and EBL cohorts. A higher CD rate was also seen in Hispanic women for the EBL cohort but not in the QBL cohort. These findings suggest that CD differences cannot explain the observed increase in blood loss and PPH in the Hispanic QBL group. Further research is needed to determine if the differences found were caused by provider cognitive bias, socioeconomic factors, language barriers and/or other factors.

This study is limited by the retrospective observational design. Only data that were easily queried through electronic medical records were collected, and free text descriptions of blood loss could not be analyzed. This original study was not designed to analyze racial and ethnic disparities or potential reasons for the differences that were found. This study was a secondary analysis and thus not powered to analyze the effect of mode of delivery as a subgroup in addition to racial and ethnic divides in blood loss measurements with EBL versus QBL. The study was performed at a tertiary care academic center with residents/fellows in a diverse major metropolitan area that may limit its generalizability. Additionally, only 10% of the parturients were African American/African and 19% were Hispanic; however, the large sample size still allowed for robust comparative analysis. As with any impact study that compares historical controls, changes in practices and patient mix may impact study results. However, the study design allowed racial and ethnic comparisons to occur concurrently in both the EBL and QBL cohorts.

## Conclusions

In conclusion, implementing a QBL system not only resulted in increased vigilance and recognition of peripartum hemorrhage, but also revealed racial disparities in peripartum hemorrhage. Racial and ethnic differences in blood loss and incidence of PPH were more apparent with objective QBL than subjective EBL assessments. Results suggest that whenever possible, blood loss should be quantified using an objective standardized instrument to avoid subjective human error and implicit bias. Future research is needed to determine what factors (e.g. provider cognitive bias, socioeconomic, language barriers) may account for racial and ethnic differences in blood loss assessments.
